# Promoter prediction in *E. coli* based on SIDD profiles and Artificial Neural Networks

**DOI:** 10.1186/1471-2105-11-S6-S17

**Published:** 2010-10-07

**Authors:** Charles Bland, Abigail S Newsome, Aleksandra A Markovets

**Affiliations:** 1 Department Natural Sciences and Environmental Health, Mississippi Valley State University, 14000 Hwy 82 West, Itta Bena, Mississippi, 38941, USA

## Abstract

**Background:**

One of the major challenges in biology is the correct identification of promoter regions.  Computational methods based on motif searching have been the traditional approach taken.  Recent studies have shown that DNA structural properties, such as curvature, stacking energy, and stress-induced duplex destabilization (SIDD) are useful in promoter prediction, as well.  In this paper, the currently used SIDD energy threshold method is compared to the proposed artificial neural network (ANN) approach for finding promoters based on SIDD profile data.

**Results:**

When compared to the SIDD threshold prediction method, artificial neural networks showed noticeable improvements for precision, recall, and *F*-score over a range of values.  The maximal *F*-score for the ANN classifier was 62.3 and 56.8 for the threshold-based classifier.

**Conclusions:**

Artificial neural networks were used to predict promoters based on SIDD profile data.  Results using this technique were an improvement over the previous SIDD threshold approach.  Over a wide range of precision-recall values, artificial neural networks were more capable of identifying distinctive characteristics of promoter regions than threshold based methods.

## Background

Identification of promoters is an important issue in biology, given that they are central in understanding the process by which genes are regulated.  Wet-lab methods for promoter identification provide accuracy but suffer from being time-consuming.  In order to facilitate faster processing, computational methods are required.  Although far from perfect, they do provide a means for quickly identifying potential targets for experimental validation.

Several computational methods for promoter prediction have been proposed. Most include some analysis of patterns commonly found in promoter regions, such as -10 and -35 motifs [1,2].  However, these patterns are not always sufficiently conserved to allow for adequate prediction.  Furthermore, there are clearly other factors not directly related to sequence motifs that are closely associated with promoter regions.  

Recent studies have reported impressive results using DNA structural properties as predictors of promoter regions [3].  These methods include DNA curvature [4], relative stability [5], and stress-induced duplex destabilization (SIDD) [6,7].  Of interest in this paper is promoter prediction using SIDD properties.  In [7], some of the most impressive results to date are reported for *E. coli* K12.  These were achieved simply using a minimum SIDD threshold value for distinguishing promoters from non-promoters.  The current study proposes a more sophisticated approach, involving the use of artificial neural networks (ANNs), along with SIDD profiles, for promoter prediction. 

## Results and discussion

A comparison of threshold and ANN methods for SIDD-based promoter prediction was assessed using *E*. *coli* K12.  First, the SIDD profile for the *E*. *coli* K12 genome was obtained from Benham [6].  For this dataset, each base pair (bp) is represented by its destabilization free energy, *G*(*x*).  *G*(*x*) corresponds to the incremental free energy needed for the base pair at position *x* to always remain open.

### Training and testing sets construction

**Figure 1:**
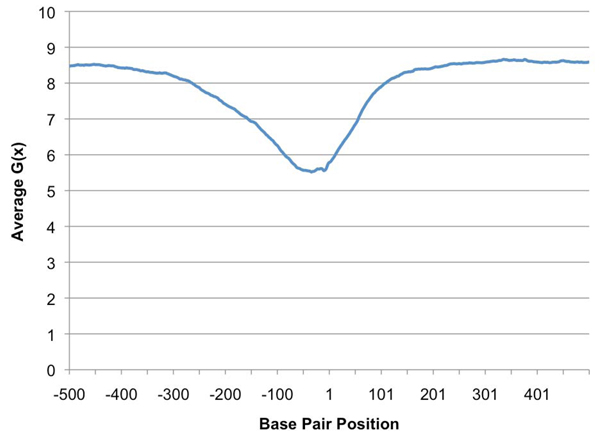
**Average G(x) for *E*. *coli* K12** The average *G*(*x*) in the range -500 to +501, relative to known transcription start sites, for *E*. *coli* K12.

The training/testing dataset was constructed from the SIDD profile.  Positive instances (promoters) were defined as the 250 bp region from -200 to +50, with respect to known transcription start sites (TSSs).  This range covers the areas of lowest SIDD energy levels surrounding promoters regions, as shown in Figure 1.  The negative instances were randomly selected 250 bp regions, excluding any located within the previously defined promoter regions.  This dataset was composed on 1648 positive instances and 4944 negative instances.  A randomly selected two-third and one-third split was used for training and testing sets, respectively.

### Comparison of prediction methods

Promoters are strongly associated with regions of low SIDD energy (see Figure 1).  This forms the basis for prediction by thresholds.  For this method, the sum of the SIDD energies, *sumG*, for each of the 250 bp testing set instance was calculated.  Instances with *sumG* less than or equal to a selected threshold, *T*, were tagged promoters.  For predictions using the ANNs, classifiers were built from the training set and evaluated on the testing set.

**Figure 2:**
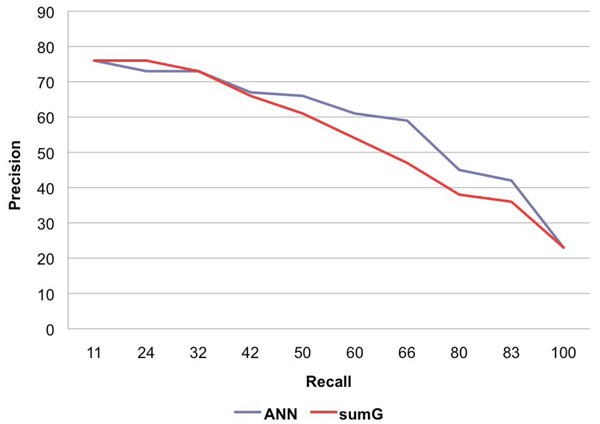
**PRC curves for ANN and *sumG* methods** The precision-recall curves for the ANN and *sumG* methods.

Comparison of ANN promoter prediction with that of prediction using *sumG* thresholds was based on precision and recall.  The first evaluation made use of precision-recall curves (PRCs), which plot recall against precision over a range of values.  The PRC for *sumG* was derived from predictions at varying thresholds.  Generating a series of precision-recall pairs for the ANNs was not as straightforward, as there was no single variable that could be adjusted in order to produce somewhat predictable precision-recall results.  The ANN application that was used did, however, provide a means of specifying costs for true positive, true negative, false positive, and false negative predictions.  By varying the costs for these measures, several artificial neural networks were produced and corresponding precision-recall pairs were used to produce the PRC shown in Figure 2, along with that of *sumG*.  Note that recall distances on the *x*-axis are not equal due to the difficulty in producing exact recall values from classifiers, particularly neural networks.   

Predictions were impressive for both classifiers and show that SIDD can be useful in distinguishing promoter and non-promoter regions.  With exceptions for conditions of high precision where the two classifiers were nearly equal, predictions for ANNs outperformed *sumG* over a wide range.  For example, at equal precision of 61% for both classifiers, the recall of the ANN was at 60%, while *sumG* was at 50%.  This represents 305 correctly predicted promoters for the ANN and 255 for *sumG*.

In order to compare predictions using a one-dimensional performance measure, the weighted average (or more specifically, the harmonic mean) over all precision-recall pairs was computed for the ANN and *sumG* methods.  This measure is known as the *F*-score.  The maximal *F*-score score for all precisions-recall pairs was identified.  The resulting values were 62.3 (precision 59, recall 66) for the ANN and 56.8 (precision 54, recall 60) for *sumG*.  Again, the ANN classifier showed a noticeable improvement over *sumG*.

A comparison was also made for a combined ANN-*sumG* classifier.  Using *F*-scores of 60 and 57 for the ANN and *sumG* classifiers, respectively, a decision tree was built from their combined predictions using the Weka data mining suite’s J48 algorithm [8].  The *F*-score of the resulting classifier was 60.  Because a large percentage of the correct predictions made by *sumG* were also made by the ANN, there was no significant improvement.

The differences in the prediction ability of the ANN and *sumG* methods result from the way they recognize promoter regions.  The *sumG* method is only able to identify SIDD regions whose summations fall above or below a specified threshold.  This differs from ANNs, which are known for their ability to detect patterns.  For example, Figure 1 shows the average *G(*x*)* at each position in the range from -500 to +500, relative to TSSs.  It can be seen that a particular pattern emerges.  The lowest energy values are present near the TSSs and gradually increase with distance; more so in the downstream direction.  Other noticeable patterns also appear near the TSSs.  ANNs were able to recognize differences in SIDD energy levels, as with *sumG*.  But, it is likely that their additional ability to recognize patterns such as in Figure 1, that gives them the advantage over the threshold method.

## Conclusions

DNA structural features are increasingly being recognized as an important tool for detecting promoters.  Promoter prediction based on SIDD information has shown promising results.  However, the current prediction method used is based simply on determining if the energy values of particular SIDD regions fall below a threshold.  Artificial neural networks were used to predict promoters based on SIDD profile data.  Results using this technique showed noticeable improvements over the current threshold method.

Future research will involve combining SIDD-based ANN promoter predictors with other methods.  In [7] it was shown that SIDD was not directly related to primary sequences or unique motifs, and not positively correlated with DNA curvature.  Thus, using SIDD with other predictive sequence and structural properties, particularly those not strongly correlated, may be gainful.  This was the approach taken in [7].

In addition, it may be useful to determine whether neural networks trained on one genome predict well on others.  Wang and Benham [7] noted nearly identical thresholds for SIDD summation parameters for *E*. *coli* K12 and *B*. *subtilis* predictions, when illustrating how SIDD properties could be used for estimating the probability that a DNA fragment contained a promoter.  This will become practical as SIDD profiles for other genomes become more readily available. 

## Methods

### Sequence data

The whole genome of *E. coli* K12, which contains 4,639,675 nucleotides, was downloaded from NCBI [GenBank: NC_000913.2].  Experimentally verified transcription start sites for *E. coli* K12 were obtained from the Regulon database (Release: 6.4, updated on 10^th^ August, 2009) [9]. This database release provided a compilation of 1771 promoter sequences. The dataset was filtered for unique promoters with known TSS locations, resulting in 1648 records.  The SIDD profile for *E. coli* was obtained from Benham [6] and used for constructing training and testing sets, as described below.

### Details of training and testing sets construction

The training/testing dataset was constructed from the *E. coli* K12 SIDD profile.  Positive instances (promoters) were defined as the 250 bp region from -200 to +50, with respect to filtered TSSs and with no bp at position 0.  This range covers the areas of lowest SIDD energy levels surrounding promoters regions, as shown in Figure 1.  The negative instances were randomly selected 250 bp regions, with restrictions on proximity to TSSs.  Negative instances used for the threshold method excluded any located within 250 bases of TSSs.  However, instances used for ANNs excluded only those located within 50 bases.  Allowing the neural networks to train on non-promoters that were similar to actual promoters slightly improved their ability to distinguish the two.  This dataset was composed on 1648 positive instances and 4944 negative instances, which represents a 3:1 ratio of negatives and positives.  A randomly selected two-third and one-third split was used for training and testing data, respectively.

### Details of artificial neural network promoter prediction

ANNs, included in the Tiberius v5.5.0 data mining software [10], were used to build predictive models for promoters, based on SIDD energy values. Each network was composed of three layers:  input, hidden, and output.  The input layer consisted of 250 input nodes.  This corresponds to the number of SIDD energy values from the training and testing instances.  The hidden layer was composed of two neurons, and the output layer one neuron.  The neural networks were trained on the training set, and the generated networks were exported outside of Tiberius for testing.

### Details of SIDD-based promoter prediction

Prediction by the threshold method was done by first calculating the sum of the destabilization free energies, *sumG*, for each of the 250 bp testing set instances.   *sumG* is defined as follows

*sumG* = 

where *G*(*x*) corresponds to the destabilization free energy at position *x*.  Instances with *sumG* less than or equal to a selected threshold, *T*, were tagged promoters.  Otherwise they were tagged non-promoters.

### Precision, recall, and *F*-score

Precision, recall, and *F*-score were defined as follows,

precision =  ,	   recall =  ,   *F*-score = 

where *TP*, *TN*, and *FP* are the numbers of true positives, true negatives, and false positives, respectively.

## Authors' contributions

AAM completed implementation and testing of the SIDD threshold method.  CB completed training and testing of the artificial neural networks.  ASN provided guidance and domain knowledge.  The manuscript was written jointly.

## Competing interests

The authors declare that they have no competing interests.
